# The Challenge of 3D Bioprinting of Composite Natural Polymers PLA/Bioglass: Trends and Benefits in Cleft Palate Surgery

**DOI:** 10.3390/biomedicines9111553

**Published:** 2021-10-27

**Authors:** Damien Brézulier, Louis Chaigneau, Sylvie Jeanne, Ronan Lebullenger

**Affiliations:** 1 CNRS, University of Rennes, ISCR-UMR 6226, 35000 Rennes, France; louis.chaigneau@univ-rennes1.fr (L.C.); sylvie.jeanne@univ-rennes1.fr (S.J.); ronan.lebullenger@univ-rennes1.fr (R.L.); 2 Pôle Odontologie, CHU Rennes, University of Rennes, 35043 Rennes, France

**Keywords:** biomaterials, regenerative medicine, cleft palate, bioglass, bioprinting, additive manufacturing

## Abstract

Cleft lip and palate is the fourth most common congenital malformation. Its prevalence is about 1 in 750 to 1 in 2000 live births. The consequences of this malformation are major: maxillary growth deficit, unaesthetic appearance, phonation disorders, difficulty in eating, and psycho-social disorders. Cleft palate repair establishes the division between the oral and nasal cavities. The alveolar bone graft is a key step. Different sites of autogenous bone harvesting are used, the most common being the iliac crest. Nevertheless, the large number of complications associated with harvesting has led to the use of substitute biomaterials. Bioactive glasses, discovered in 1969, are a group of synthetic silica-based materials with bone-bonding properties. Although 45S5 granular composition is commonly used in bone surgery to repair critical defects, it is only rarely used in the repair of cleft palates because this galenic form is only moderately adapted. However, advances in bone tissue engineering allow the shaping of three-dimensional scaffolds, which support colonization by host cells. Recent advances in computer-aided design/computer-aided manufacturing (CAD/CAM) have even led to the 3D printing of scaffolds combining 45S5 bioglass with a natural and biocompatible poly-lactic acid matrix. The shape of the parts is customized and adapted to the particular shape of the critical bone defects. The objective of this literature review is to highlight the particularities of alveolar defects subsequent to facial clefts, then to detail the characteristics of the materials and technologies used to elaborate 3D matrices by bioprinting. Finally, we will explore research directions regarding their use in reconstructive surgery of cleft palates.

## 1. Introduction

Cleft lip and palate are common embryonic developmental anomalies with an estimated incidence of 1 in 750 to 1 in 2000 live births [1]. It consists of an absence of bud fusion during the second month of intrauterine life. Children with a cleft palate have a lack of separation of the nasal and oral cavities. Multiple functional disorders, such as unsightly appearance and speech and eating difficulties, require early multi-disciplinary care [2]. The objective of this procedure is to establish a separation between the two cavities and to reconstruct the missing portion of the dental arch. The continuity of the upper jaw can only be established if a bone bridge is created between the two alveolar fragments resulting from the lack of embryonic fusion. This sequelae can be considered a critical size bone defect in the sense introduced by Schmitz and Hollinger: “bone defect on a particular anatomical site that does not heal spontaneously and completely over a given time” [3].

The use of bone grafts or synthetic substitutes is widely described in regenerative medicine literature [4]. More than 2.2 million bone grafting procedures are performed worldwide each year to repair bone defects in orthopedics, neurosurgery, and dentistry [5]. Faced with the therapeutic challenges in this area, a large number of substitutes have been proposed. Ideally, bone substitutes should be biocompatible, have a minimal fibrotic reaction, undergo remodeling, and promote bone neoformation. From a mechanical perspective, synthetic bone substitutes should have a strength similar to that of the cortical and cancellous bone they replace.

Bioactive silicates are already used in cleft pediatric surgery to replace the iliac crest bone harvesting. Available in granular form, they are inserted into the bone defect during surgery prior to gingival soft tissue plasty. One of the main difficulties is the stabilization of the biomaterial in this form before suturing the surgical site [6].

Contemporary CAD/CAM techniques (Computer-Aided Design/Computer-Aided Manufacturing) allow scaffolds to be shaped prior to surgical placement. Such scaffolds are easily made of composite materials, ensuring optimal biological and physical properties. Materials such as bioactive silicates associated with a poly-lactic acid matrix are suitable for bioprinting custom-made parts, easily applied to the bone defect [7]. From the X-ray imaging data, a corresponding object is designed and printed. This part must be easily manufactured and sterilized before being positioned during the operation. We are only at the beginning of the development of three-dimensional customization of bone replacement grafts. In this context, the objective of this literature review is to highlight the advances in bone tissue engineering made possible by the 3D printing of natural biomaterials and to detail the directions of research regarding their use in reconstructive surgery of cleft palates.

## 2. Facial Clefts

### 2.1. Prevalence and Etio-Pathogenesis

Cleft lip and palate are divided into two groups that differ epidemiologically and etiologically: isolated cleft palate and cleft lip with or without cleft palate. In the first case, one gene has been identified, but many more are probably involved. In the second case, several loci have been identified, and, in one case, a specific gene has also been found. It represents a heterogeneous group of disorders affecting the lips and oral cavity [8,9,10,11]. Cleft lip and palate is the fourth most common birth defect and the first most common craniofacial anomaly [1]. The incidence of cleft lip and palate varies from 1 per 750 live births to 1 per 2000 live births, depending on the geographical area [12]. Cleft palate is a feature of more than 200 well-defined congenital malformation syndromes [1]. However, nearly 70% of cases are considered non-syndromic [13]. At present, the described etiologies are multifactorial and involve genetics, environmental factors, and teratogens [14,15]. In addition, gene–environment interactions for non-syndromic clefts are numerous. Gene-environment interaction is defined as “a different effect of an environmental exposure on disease risk in persons with different genotypes”, or, alternatively, “a different effect of a genotype on disease risk in persons with different environmental exposures”. For example, the TGF-alpha gene and smoking or alcohol, nutritional factors and the MSX1 and TGFB3 genes [16,17].

The embryology of the palate has been known for a long time; its development takes place between the fourth and twelfth week of gestation. During the fourth week, the development of five facial prominences begins when neural crest cells migrate to the craniofacial region under the regulation of a number of homeobox genes [12]. The five facial prominences are the fronto-nasal, maxillary, and mandibular pairs. Molecular studies in animal models have shown the role of fibroblast growth factor (FGF), sonic hedgehog (SHH), bone morphogenetic proteins (BMP), and retinoic acid (RA), among others, in the initiation and growth of facial processes [8].

By the end of the fourth week, the nasal placodes develop. The nasal cavity develops within the placodes, with paired medial and lateral nasal processes developing from the fronto-nasal process on either side of the nasal placodes. By the end of the sixth week, the medial nasal processes fuse, forming the philtrum, and then by the end of the eighth week, they fuse with the adjacent maxillary processes, forming the upper lip and primary palate anterior to the incisive foramen. Prior to fusion, the nasal processes undergo a peak in cell division, resulting in increased susceptibility to teratogenic stress and an increased risk of fusion failure. Fusion failures at this stage result in cleft lip with or without palatal division [18].

At the same time, between the sixth and ninth week, the lateral palatal processes fuse after the secondary palate has been brought closer to the primary palate with a junction marked by the incisal foramen [10]. Multiple genetic (e.g., mutation on IRF6, MAFB, ABCA4 genes) and environmental factors (e.g., maternal smoking, maternal alcohol, nutritional factors, radiations) influence the growth, approach, and fusion of the fronto-nasal and maxillary processes [19]. These factors can lead to local changes in growth factors, extracellular matrix (ECM) and cell adhesion molecules to the point of causing a lack of fusion processes, leading to secondary cleft palate [20].

### 2.2. Current Care and Support

Although it is not yet possible to completely prevent the occurrence of cleft palates, they have a substantial impact on the quality of life of children and adolescents [21,22]. They have major consequences on the jaw structure, aesthetic appearance, speech, eating, and swallowing. They can be addressed and corrected appropriately by the intervention of an interdisciplinary team [2]. Beyond the anatomical aspect, it is also in charge of the psychological care of the affected children. The work carried out covers the themes of family adaptation, appearance, self-esteem, social interaction, emotional and behavioral adaptation, and cognitive functioning [23].

Cleft palate repair establishes the division between the oral and nasal cavities. Suitability for palatal repair, timing, and surgical method of repair depend on comorbid conditions and are subject to discussion [24]. The objectives of palatoplasty are the separation of the nasal cavity from the oral cavity, the creation of a velo-pharyngeal valve competent for speech and swallowing, and the preservation of the growth of the middle face. For this purpose, it is recommended to perform it before 12 months of age, ideally from 3 to 6 months of age, i.e., before the acquisition of language begins [25]. There are multiple surgical techniques such as those of Von Langenbeck, Veau-Wardill-Kilner, or the two-part palatoplasty [26].

The next step occurs in the mixed dentition stage. This key stage reconstructs the continuity of the alveolar bone. It provides support to the maxillary arch for dentition and mastication [27,28]. An orthodontic expansion period is a prerequisite for a surgical procedure of labial, gingival, and osseous reconstruction [24,29]. Simple surgical closure is insufficient and requires the addition of materials to fill these critical defects. The average volume of the alveolar cleft is about 620 mm^3^ [30]. The use of bone grafts or synthetic substitutes is described [4]. These materials can be of natural origin, such as autografts or allografts. Different sites for autogenous bone harvesting are used: the most common is the iliac crest, but the tibia, mandible, rib, and skull can also be used [31,32]. However, the high resorption rate during the first year after grafting is the first limitation [33]. The second limitation is the complications related to harvesting: postoperative pain, difficulty in walking, nerve damage, hematomas, infections, and arterial wounds have been described [34,35,36,37,38,39].

In this context, alternatives to autologous bone grafting have been sought [40,41]. Synthetic substitutes such as hydroxyapatite (HA), tricalcium phosphate (TCP), bioactive silicates (SiO_2_), lactic or glycolic acid polymers (PLA and PGA), or biocomposites constructed from combinations can be used [42]. Bone substitutes such as bioactive silicates are already used in pediatric surgery to replace the iliac crest bone harvesting [6,43,44]. Applied to the field, the 45S5 bioglass brings promising results with 80% of bone continuity at one year in a study on 58 cases [6].

## 3. Bioglasses

### 3.1. A Few General Points

**Figure 1:**
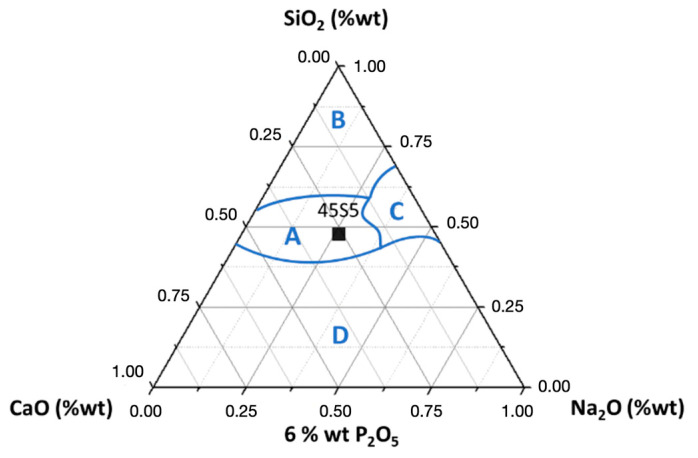
Hench diagram and bioactivity index in the SiO_2_-Na_2_O-CaO system and 6% P_2_O_5_. 45S5 refers to the composition of bioglass according to Hench description.

Bioactive glasses are a group of synthetic bioactive inorganic silica-based materials with bone-bonding properties, first discovered by Larry Hench. The first bioactive glass was synthesized in 1969 and belonged to the SiO_2_-Na_2_O-CaO-P_2_O_5_ system. He found that certain glass compositions had excellent biocompatibility as well as the ability to bind to bone [45]. Extending these experiments, he deepened the SiO_2_-Na_2_O-CaO-P_2_O_5_ quaternary system by keeping the mass fraction of P_2_O_5_ constant and equal to 6%. He deduced the so-called Hench diagram, which shows four zones (Figure 1).

Zone A is called the bioactivity zone: chemical bonds at the glass/bone interface are created in less than 30 days. Zone B (bio-inertia zone) is marked by a too low reactivity leading to the formation of a fibrous capsule that inhibits the bond between the bone and the glass. Zone C, the resorbability zone (too high reactivity), leads to total resorption of the bioglass in less than 30 days. Zone D does not lead to homogeneous glasses (devitrification or phase separation). The results of this work show that the critical characteristic for the rate of bioactivity is a silicate SiO_2_ content that must be maintained below 60% of the weight.

Hench recently published the history of bioactive glass development with emphasis on the discovery of the “classic” 45S5 composition [46]. This oldest composition consists of a silicate network (45 wt% SiO_2_) incorporating 24.5 wt% Na_2_O, 24.5 wt% CaO, and 6 wt% P_2_O_5_ [47]. It should be noted that for many years, other biocompatible glass compositions have been synthesized, such as Bioglass 13–93 (comprising K_2_O and MgO) or Bioglass S53P4 (including Fe) [48,49,50]. The preferred use of certain bioglass compositions depends on their applications.

### 3.2. Shaping

**Figure 2:**
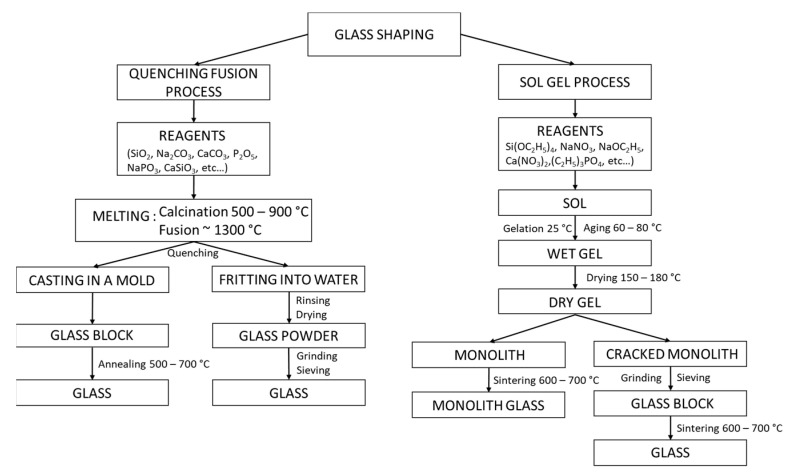
Comparative diagram of quenching fusion process and sol–gel process.

Currently, two preferred processes are used for glass synthesis: the melt-quench method and sol–gel technique (Figure 2).

#### 3.2.1. Shaping by Quenching Fusion Process

Commonly, and for several millennia, the fusion of a mixture of several oxides followed by a quenching has allowed to obtain a bioglass [51]. The precursor oxides and/or carbonates are weighed in the desired proportions, ground and mixed to obtain a homogeneous powder. The mixture is deposited in an alumina crucible for low-temperature glasses (alumina diffuses at high temperatures) or in platinum [52], then placed in a high-temperature oven (>1300 °C). The powders are calcined at 500 to 900 °C, depending on their composition, for at least 90 min to remove gaseous substances from the mixture of oxides and/or carbonates [53]. The mixture is then brought to melting temperature (up to 1500 °C for some glasses) and maintained for one hour to obtain a homogeneous molten mixture. Two options are then possible: obtaining a voluminous glass with a particular geometry or a cluster of small grains.

For the first option, the liquid mixture is poured into a mold preheated at Tg (glass transition temperature) and then placed in an oven at Tg for 1 h. This last step allows to shape the liquid mixture while limiting the presence of intrinsic mechanical constraints inside the glass [54]. The size and shape of the mold used determine the macroscopicity of the glass. For the second option, the molten mixture is quenched in a cold-water bath. The thermal shock to the glass causes very high mechanical stresses, which break the glass. The resulting grains are often called “frits”. They are easily reduced to powder.

#### 3.2.2. Shaping by Sol–Gel Process

The “solution gelification” technique, usually called the sol–gel technique, is a synthesis method of materials (glasses, ceramics, or organo-minerals) from precursors in solution. As a reminder, a sol is a colloidal solution where fine particles (1–1000 nm) are suspended in a liquid, whereas a gel is a three-dimensional network of macromolecules trapped in a liquid. Unlike the melt-quench process, the sol–gel technique uses a low temperature (<300 °C). This solution is contemporary: the first polymerization was performed by J.J. Ebelmen in 1845.

The elaboration of bioglass by sol–gel process has since been widely studied [55]. Oxide networks are obtained by solution polymerization of silicon alkoxide precursors Si(OR)n, where OR corresponds to a deprotonated alcohol. For the other elements, desired in the final composition of the glass, (C_2_H_5_)_3_PO_4_, (Ca(NO_3_)_2_-4H_2_O), (NaNO_3_), ((MgNO_3_)_2_-6H_2_O) and (KNO_3_) can be used as a source of P_2_O_5_, CaO, Na_2_O, MgO and K_2_O [56]. Polymerization involves two steps: hydrolysis and condensation [57]:

− Hydrolysis: Si-OR + H_2_O → Si-OH + ROH
− Condensation: Si-OH + RO-Si → Si-O-Si + ROH or Si-OH + HO-Si → Si-O-Si + H_2_O

The commonly used silicate precursors are tetraethyl and tetramethyl orthosilicates (TEOS, TMOS). Silicon alkoxides are not miscible with water, so the reaction is facilitated by the use of a co-solvent, often an alcohol. The obtained nanoparticles are then dried and calcined to form bioglass nanoparticles.

The sol–gel method therefore offers an interesting alternative to the quenching fusion technique. The sol–gel method thus offers an interesting alternative to the quenching fusion technique. For example, it allows to obtain bioglass nanoparticles and not a block. By modifying the experimental conditions, it is also possible to control a number of structural parameters. Zheng and Boccaccini have summarized the different methods for producing nano-bioglass and their specific characteristics [57]. For example, using Stöber’s method, in basic catalysis, the size [58,59] and morphology [60] of the nano-bioglass can be controlled by varying the following parameters: ethanol/H_2_O ratio, pH, reaction temperature, and synthesis pressure. In addition, Stöber’s method allows the synthesis of porous materials with controlled pore size and shape. Controlling the concentration of cetyltrimethylammonium bromide (CTAB) allows the production of lamellar, hexagonal, or radial pores [60,61]. The template used governs the spatial arrangement of the pores: hexagonal with pluronic F127 [62] and radial [63] with cetylpyridine bromide (CPB).

### 3.3. Biological Properties of Binding to Bone

Bioglass 45S5 bonds rapidly to bone. It is attributed to the formation of a hydroxyapatite (HA) layer that interacts with collagen fibrils in damaged bone. It also has the ability to stimulate bone growth away from the bone–implant interface. A hydroxyapatite layer is formed as a result of the dissolution of glass by interstructural fluids, in a mechanism similar to that of conventional glass corrosion [64]. Dissolution is responsible for creating surface sites and a pH suitable for the nucleation of hydroxyapatite crystals. The formation of the hydroxyapatite layer in biological fluids is described in five steps [65]:

- Rapid exchange of Na^+^ and/or Ca^2+^ cations with H^+^ from the solution, creating silanol (Si-OH) bonds on the glass surface;
- Attack of the silica glass network by OH^−^;
- Condensation of Si-OH groups near the glass surface: re-polymerization of the silica-rich layer;
- Migration of Ca^2+^ and groups to the surface through the silica-rich layer and from the solution, forming an amorphous CaO-P_2_O_5_-rich film on the silica-rich layer;
- Incorporation of hydroxyls and carbonate from solution and crystallization of the CaO-P_2_O_5_ film into hydroxyapatite.

**Figure 3:**
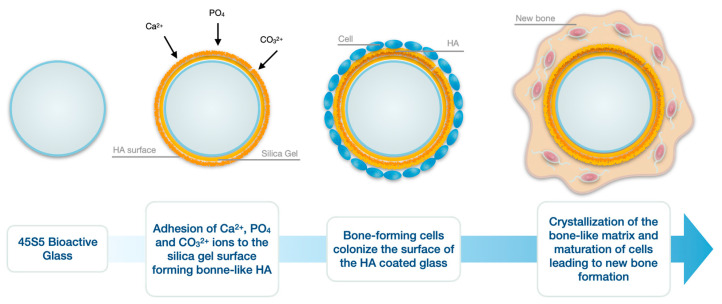
Biological reaction following the insertion of a bioglass into a bone defect. The reaction with physiological fluid is illustrated in the first two steps. The formation of new bone is illustrated in the last two steps. HA: hydroxyapatite.

Once the HA layer has formed, binding to the bone begins. The cells quickly attach to the growing HA layer and begin to multiply and differentiate into osteoblasts. These highly specialized bone cells produce a unique protein, called type 1 collagen, which nucleates the crystalline HA platelets within the collagen fibrils. The osteoblasts become enclosed in the HA-enhanced collagen layer and develop into a mature bone cell, called an osteocyte, which is unable to divide [66] (Figure 3).

## 4. Poly-Lactic Acid Matrices, Natural Polymers

### 4.1. Overview

Poly-lactic acid (PLA) is a linear aliphatic polyester derived from lactic acid (2-hydroxypropionic acid). Fermentation of lactic acid from sugars produces PLA polymers, which makes them ecological and eco-friendly [67,68]. In 1970, the US Food and Drug Administration (FDA) approved PLA for use in direct contact with biological fluids. Today, it is used in many medical applications because of its biocompatibility and degradability in the human body. The biodegradation of PLA-based polymers involves their hydrolysis into lactic acids which are finally eliminated from the body in the form of CO_2_ and H_2_O [69]. PLA is the perfect example of a “biomaterial”. A biomaterial can be defined as “any material intended to be in contact with living tissue and/or biological fluids and having the function of evaluating, treating or replacing any tissue, organ or function of the body”. PLA has innovative multidimensional applications [70,71]; as a carrier for the progressive release of a drug by hydrolytic degradation and/or by morphological modifications of the polymer [72]; as interference screws for the ankle, knee and hand or as pins for ligament fixation [73]; or even in tissue engineering. In this last case, used alone or in combination with other biodegradable polymers, it constitutes a support for cell growth. It is then used in different forms: filamentary, braided, knitted, or as a film.

**Figure 4:**
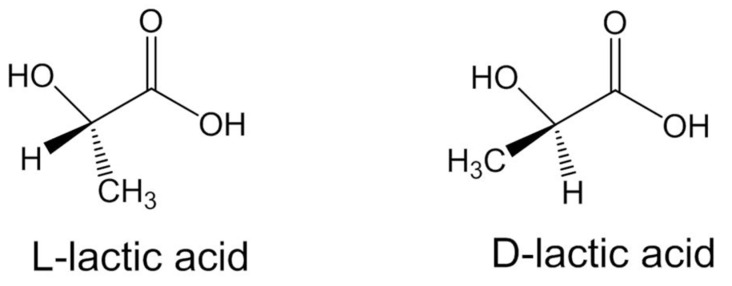
Enantiomeric structures of poly-lactic acid monomer. Polymers containing exclusively either (L)-lactic acid (PLLA) or (D)-lactic acid (PDLA) are semi-crystalline with a glass transition temperature (Tg) of about 60 °C and a melting temperature (Tm) of 175 °C. Semi-crystalline PLA has a Young’s modulus of between 3 and 3.5 GPa, a tensile strength of between 50 and 70 MPa.

The frequent use of these polymers is due to the fact that they offer good bone fixation because it is possible to adapt the rate and degree of crystallinity and, consequently, the mechanical properties, degradation behavior, and processing temperatures [74]. These results are obtained by controlling the stereochemical architecture and the molecular weight of the polymer. The two stereoisomers of PLA are PLLA (poly(L-lactide)) and PDLA (poly(D-lactide)) (Figure 4). PDLA is amorphous; its erosion occurs at a much faster rate than PLLA. In comparison, highly crystalline PLLA will take 2 to 5 years to degrade in a normal physiological environment, while amorphous PDLA will show a loss of integrity in 2 months and complete degradation in 12 months under similar conditions [75].

### 4.2. Shaping and Sterilization

Before producing PLA, let us study its precursors (lactic acid monomers and lactides). They come from the fermentation of renewed agricultural sources. They are therefore produced at low costs compared to other similar polymers. Lactic acid is a 2-hydroxycarboxylic acid with two stereoisomers: L-lactic acid (levogyre) and D-lactic acid (dextrogyre). They make it possible to obtain different lactides. Lactide is obtained from depolymerization of oligo(lactic acid), itself obtained by the polycondensation of lactic acid, catalyzed by metal compounds like zinc (Zn) or aluminum (Al). The lactide is then recrystallized by solution.

In general, two methods are used to obtain PLA: (i) the polycondensation of lactic acid and (ii) ring-opening polymerization of lactide. (i) Polycondensation of lactic acid was first studied in 1995 using azeotropic dehydration of lactic acid and the catalyst in an aprotic solvent [76]. To produce high-molecular-weight PLA, it is essential to run the process under reduced pressure to minimize water in the polycondensation [77]. (ii) Ring-opening polymerization is a technique for controlling the molecular weight of PLLA and thus obtaining a high molecular weight. Manufacturers, such as NatureWorks LLC (a major PLA producer), mainly use it for high-throughput production.

Polymerising L-lactide or D-lactide results in either PDLA or PLLA. As these two stereoisomers do not have the same properties, it is possible to adjust the use of polymers according to their applications [78].

Commercial PLA is commonly supplied in the form of millimeter-sized granules. However, depending on the application, it is sometimes necessary to reduce the granulometry or to transform them into a filament. To obtain the latter, using a filament extruder is the most trivial method. The pellets are deposited in a hopper, and an auger feeds the pellets into a nozzle heated to the extrusion temperature of PLA, in the same way as a 3D printer. However, to obtain a homogeneous filament, it is important to control the extrusion rate. For this purpose, machines exist that allow the filament to be wound at a constant speed, thus controlling the extrusion rate. To reduce the size of the granules, many methods exist such as single emulsion, double emulsion, nanoprecipitation, etc. Lee et al. presented all theses techniques [79].

For any in vivo use, PLA-based constructs must be sterilized. Sterilization ensures patient safety without compromising the polymer structures, physicochemical properties, biocompatibility, morphology, or topography. The literature shows that these properties are retained when gamma radiation is used to sterilize PLA electron beam, ethylene oxide, and hydrogen peroxide [80]. Conversely, saturated steam sterilization leads to profound changes, even damaging the chemical structure of PLA (morphological, physical, and thermal changes resulting from the pressure and high temperature) [81]. The use of PLA as an implantable material therefore requires prior physicochemical characterization tests. The same applies to the use of PLA to make a composite material.

## 5. Shaping by 3D Printing for Biomaterials

Since the first patent on stereolithography was filed in 1986, the additive manufacturing sector has been expanding rapidly. Indeed, different techniques have been successively developed to respond to the problems of complex shaping of certain materials. The use of CAD (Computer Aided Design) software makes it possible to free oneself from the geometric constraints induced by the use of a mold. Processes such as fused deposition modeling (FDM), robocasting, powder bed printing, or stereolithography are processes are mainly used in industry [82]. With the emergence of these new technologies, it would seem trivial to form a glass with a complex geometry using these processes. However, due to its particular physicochemical properties, little progress has been made in the 3D printing of glass materials. The need to provide a significant amount of energy to melt the glass, combined with its variation in viscosity depending on temperature, makes glass a difficult material to produce by additive manufacturing. However, some experiments have been carried out successfully, such as the printing of chalcogenide (Tg = 150 °C) [83,84] and phosphate (Tg = 250 °C) glasses [85] by FDM or silicate glasses by stereography [86] and selective laser melting [87].

To produce implants with complex geometry, research was therefore very quickly directed towards the production of glass–polymer composites. Conclusive assays were carried out on various polymers such as poly(e-caprolactone) (PCL) [88] or polyetheretherketon (PEEK) [89]. However, due to its low cost, high abundance, biocompatibility properties, and faster degradation rate, PLA is the most studied composite matrix in the field of bone regeneration.

**Figure 5:**
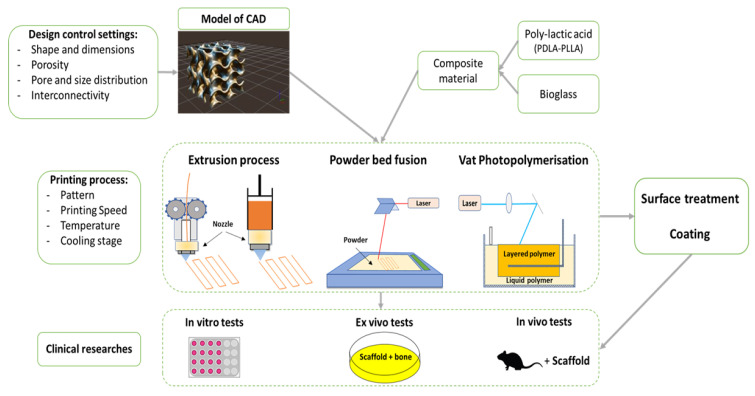
Summary diagram of the different steps in the development of scaffolds as biomaterials.

The primary objective of 3D printing in the medical field is to optimize the mechanical properties of scaffolds while ensuring appropriate porosity for cell attachment and proliferation. Moreover, the maximum pore resolution of the objects obtained in printing is at most 10 μm [90,91]. In addition, the obtained 3D parts usually have low biological activity, requiring post-printing surface treatments [92,93]. A summary diagram showing the different steps required to manufacture a PLA-bioglass scaffold is shown in Figure 5.

### 5.1. PLA–Bioglass Composite

As mentioned in the previous paragraphs, bioglasses and PLA are biocompatible compounds that can be inserted into the human body. For this reason, several studies have been carried out to assemble them. In order to perfect the bioactivity of the composite and to strengthen the tissue-PLA bond, different methods have been implemented. We can mention the modified phase separation protocol [94], the pre-treatment of bioglass with polydopamine [95] or the coating of PLA/chitosan composite with bioglass [96]. In addition, biological tests have been undertaken to confirm the capacity of the composite to serve as bone tissue replacement (bioactivity, cell cultures, in vitro degradation, etc.) [97,98,99,100,101,102]. SBF tests lasting 28 days were also performed on composite scaffolds which validated their ability to produce hydroxyapatite on their surface.

To optimize the long-term degradation of the polymer, it is important to strengthen the polymer–bioclay interface [99]. To achieve this, it is possible to treat the bioglass with 3-ami-nopropyltrimethoxysilane (APS) [103], a chemical with the ability to join organic and non-organic phases, and thus strengthen the interface, or by cross-linking dopamine on the surface of the bioglass [95].

### 5.2. Vat Photopolymerisation

Vat photopolymerisation is an additive manufacturing technique based on the layer-by-layer production of a curable material when subjected to radiation (laser or digital light projection) [104]. The initial monomers, which are sensitive to the photon’s radiation, become radicalized and cause a chain reaction to polymerize and thus solidify each layer. To obtain a PLA-based matrix, the first step is to allow its oligomers to become thermocurable. To do this, they react with an initiator, such as hexanediol [105], to obtain a hydroxyl group at the end of the chain. They are then mixed with a reagent allowing them to acquire methacrylate groups. The photosensitive species must then be diluted in a solvent to reach the liquid state before radiation exposure.

However, the choice of solvent poses an important problem. Solvents can become reactive, interact, and ultimately end up in the final composition of the matrix. To counter this phenomenon, it is possible to use non-reactive solvents which can then be washed with biocompatible liquids and thus avoid any trace of the solvent during the subsequent biological steps. However, as Melchels et al. have shown, the use of such non-reactive solvents can have significant consequences on the final appearance of the piece [106].

It is also possible to produce matrices of several polymers containing PLA. For example, photopolymerisation tests have been carried out on a resin based on the monomer PDLLA-PEG-PDLLA [107]. They showed good cell compatibility and soft tissue-like mechanical properties.

As demonstrated by Arcaute et al. with a Poly(Ethylene Glycol) matrix, one of the main advantages of PLA is the ability to control the placement of cells and bioactive agents in the scaffold during part construction [108]. However, PLA is probably not the most suitable biopolymer for this technique.

### 5.3. Powder Bed Fusion

There are three main techniques in powder bed fusion: SLS (Selective Laser Sintering), SLM (Selective Laser Melting), and three-dimensional printing (3DP). As there are almost no publications on PLA, the latter will not be discussed. The process that combines SLS and SLM involves applying a laser to a layer of powdered material deposited on a substrate [109]. This results in sintering/fusing of the powder particles so that they are locally sintered/fused together. This step is carried out successively for the different layers of the object. Under the effect of the laser, the n-1 layers will sinter/fuse with the n layer. Once the 3D object has been designed, the excess powder is evacuated by a vacuum. Various treatments such as sintering, polishing, coating, or infiltration can then be applied to the final piece to modify its properties. Different laser wavelengths can be used depending on the absorbency properties of the material. The most common are YAG (λ~1 μm), CO (λ~5.5 μm) and CO_2_ (λ~10 μm) lasers. The main parameters involved in this process are the power and speed of the laser, the distance between two successive applications (important for resolution), and the particle size of the powders used. Three-dimensional metal objects, notably in titanium, have already been designed by the powder bed fusion method for supporting applications in regenerative medicine [110].

For biopolymers, the SLS technique sometimes causes numerous problems. Indeed, the high process temperature does not allow the incorporation of cells during the scaffold building. In addition, it can also lead to chain scission, cross-linking, or oxidation, which can cause polymer degradation [97,111]. Williams et al. show that the use of PLA as a bone regeneration matrix in SLS is limited compared to other polymers such as PCL [112]. Indeed, to maximize the rendering of the final part, it is important to use a fine-grain-size powder. However, to obtain a nanoscale powder, most of the techniques used include a step using toxic solvents such as dichloromethane or chloroform, which are incompatible with medical applications. Mechanical grinding at low temperature (T < polymer Tg) is possible, but generally does not allow regular shaped particles to be obtained, which are essential to obtain a suitable density for the material [113].

However, a few papers demonstrate the feasibility of the process. Interesting results were shown by Duan et al., who produced calcium phosphate–PLLA composites [114]. After printing, high viability was obtained after 3 days for SaOS-2 cells and their attachment and proliferation after 7 days. Simpson et al. have also developed a poly(L-lactide-co-glycolide)/hydroxylapatite composite in SLS with mechanical properties similar to trabecular bone [115]. This material could probably be considered as an alternative supporting construction.

### 5.4. Extrusion Techniques

Ordinary material extrusion techniques such as fused deposition modeling are the most common 3D printing techniques. They are inexpensive and easily implemented. The principle of this process is basic [116]. A filament of any material is fed to an extruder. The filament is fed step by step through a guide to a heated extrusion nozzle to be melted. The speed of the extruder controls the amount of material fed to the nozzle. The molten material is deposited on a substrate. The layers are deposited successively, adhering to each other, and cooled with a fan. It should be noted that the printing plate can be heated beforehand to allow better adhesion of the first layer of material. However, despite its many advantages, extrusion techniques often offer a lower resolution than other additive manufacturing techniques [117].

For bioprinting, this technique does not allow the insertion of cells before extrusion. The use of PLA as a polymer matrix, although considered low temperature (~200 °C), instantly destroys the cells. The only possibility of depositing cells in the PLA matrix composite is therefore post-impression. It is then necessary to carry out initial biological tests to determine whether the cells studied are likely to cling to the PLA composite and proliferate. In addition, when printing the composite, it is important to limit the shear stresses as they can affect the degradation properties of PLA [118].

In the early 2000s, a new generation of industrial extrusion processes emerged, namely bioprinters [119]. The first applications included the development of vascular tissue networks to hold cells in culture [120] and the production of synthetic biocompatible cell carriers [121], later called scaffolds. Extrusion-based bioprinters mainly use the 3DF (three-dimensional fiber deposition) technique. Unlike conventional 3D printers, this technique uses air pressure instead of rollers to force solutions or melts through a nozzle. Thus, it does not require a post-seeding process. Furthermore, compared with other bioprinting processes, extrusion bioprinting has many advantages [122]. It allows the use of a wide range of biomaterials and cells, including native and synthetic hydrogel polymers, cell aggregates, and decellularised extracellular matrix, whereas other techniques are limited to the bioprinting of hydrogel polymers with cells in suspension [123]. Furthermore, the deposition of biomaterials with physiological cell density, which is difficult to achieve with other bioprinting techniques, is possible with extrusion bioprinting [124].

Recently, successful tests have been performed on a bioprinted hydroxyapatite PLA scaffold loaded with enhanced bone marrow, combined with an induced membrane to repair large radial defects in rabbits [125]. These tests showed that the induced membrane combined with the PLA-HA scaffold provided the same efficacy as the induced membrane/liac crest bone graft by radiography, micro CT, and histological evaluation. Jain et al. also demonstrated the value of extrusion bioprinting by producing scaffolds from poly(L-lactide-co-trimethylene carbonate) matrices to address the problems of adipose tissue regeneration in large volume defects [126]. Stem cells, derived from human adipose tissue, attached to the scaffold and proliferated. The printing of bioglass 45S5 in a PCL matrix was also developed by Vallejos et al. [127], confirming the feasibility of bioprinting bioglass–polymer composites for application in regenerative medicine.

## 6. Application Perspective in Regenerative Medicine and Cleft Surgery

Bone defects left by clefts are real therapeutic challenges. The filling of these critical defects is facilitated by the use of bone tissue engineering techniques. The ideal material should be biocompatible, osteoconductive, osteoinductive, osteogenic, resorbable, or degradable and have mechanical properties similar to those of the bone at the implant site to provide temporary mechanical support [128]. Moreover, the possibility of shaping a scaffold with dimensions perfectly adapted to those of the bone defect is a major advantage. Digital data flows collected directly from the patient, associated with CAD/CAM technology, now make it possible to shape these scaffolds prior to their surgical placement [118]. Here, we propose a data processing workflow leading to the printing of a custom scaffold for filling of an alveolar defect sequelae of a cleft lip and palate. To our knowledge, this type of approach has not been described in clinical routine.

**Figure 6:**
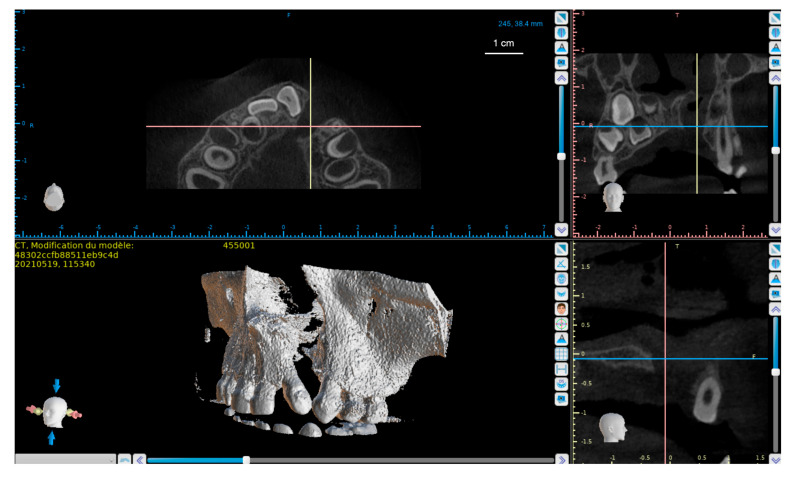
**Top left**: coronal section of a CBCT of a patient with a cleft alveolar-palate. The two fractures are perfectly individualized. **Bottom left**: volume modeling from DICOM files before export.

In a first step, the DICOM (Digital Imaging and Communications in Medicine) data acquired during the CT (Computed Tomography) cone beam is exported to the Blue Sky Plan 4 dental planning and design software (Blue Sky Bio, Libertyville, IL, USA). This software allows the selection of the region of interest, the maxilla in its dento-alveolar region in this case. Based on thresholding, only the dental and bone structures are kept. They are used to generate a three-dimensional object file in STL (Standard Triangle Language) [129] (Figure 6).

**Figure 7:**
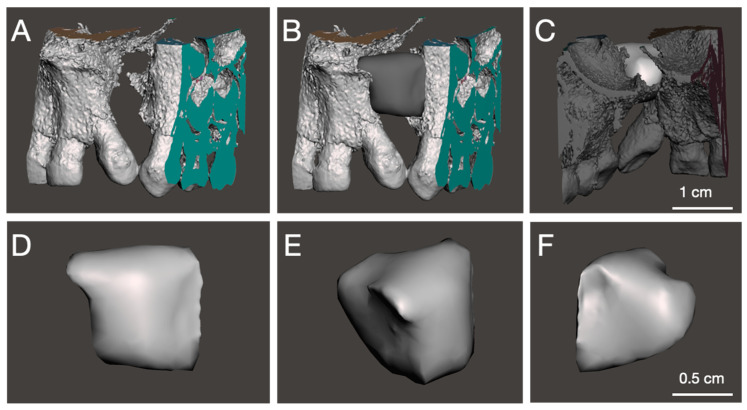
Example of design of a part from the CBCT seen in the previous figure. Initial situation (**A**) then with the part filling the critical defect in front (**B**) and posterior (**C**) view. Front (**D**), anterior (**E**), and posterior (**F**) views of the part.

In the second step, all stl data were imported into the Autodesk MeshMixer computer-aided design software [130,131]. The areas adjacent to the defect are isolated and smoothed. Then, using the extrusion function, the two surfaces are transformed into penetrating volumes. The Bollen fusion of the two elements allows to obtain a unique volume, which is smoothed. Finally, the fit on the initial stl is checked and the undercuts that prevent the insertion of the part are removed (Figure 7).

The stl file is then sent to a slicing software such as Cura and is transformed into gcode format, the format used by 3D printers. In the slicing software, it is possible to control the printing parameters to optimize the quality of the 3D part (printing speed, nozzle temperature, placement of supports, filling of the part, etc.).

## 7. Conclusions

The research and development of new materials for medical applications is a rapidly growing field. Biomaterials are an interface between chemistry, physics, and medicine, with the challenge of replacing living organisms. Natural or synthetic materials have been used for a long time. However, the last few years have seen the rapid development of CAD/CAM systems based on 3D imaging of the patient. They open up a new area for the shaping and use of biomaterials offering a multitude of therapeutic approaches to clinicians. The literature review proposed here shows how, from bioglass shaped by different techniques, it is possible to print scaffolds of materials with controlled size, strength, and porosity parameters.

Facial clefts are sequential defects of embryonic growth. To date, there is no consensus on surgical management. Substitute materials can bypass the hassle of autogenous bone harvesting. Future years will have to perfect the materials available to surgeons in order to facilitate their implementation and to perpetuate the results of the treatments.
